# Re-conceptualizing stress: Shifting views on the consequences of stress and its effects on stress reactivity

**DOI:** 10.1371/journal.pone.0173188

**Published:** 2017-03-08

**Authors:** Jenny J. W. Liu, Kristin Vickers, Maureen Reed, Marilyn Hadad

**Affiliations:** Department of Psychology, Ryerson University, Toronto, Ontario, Canada; Technion Israel Institute of Technology, ISRAEL

## Abstract

**Background:**

The consequences of stress are typically regarded from a deficit-oriented approach, conceptualizing stress to be entirely negative in its outcomes. This approach is unbalanced, and may further hinder individuals from engaging in adaptive coping. In the current study, we explored whether negative views and beliefs regarding stress interacted with a stress framing manipulation (positive, neutral and negative) on measures of stress reactivity for both psychosocial and physiological stressors.

**Method:**

Ninety participants were randomized into one of three framing conditions that conceptualized the experience of stress in balanced, unbalanced-negative or unbalanced-positive ways. After watching a video on stress, participants underwent a psychosocial (Trier Social Stress Test), or a physiological (CO_2_ challenge) method of stress-induction. Subjective and objective markers of stress were assessed.

**Results:**

Most of the sampled population regarded stress as negative prior to framing. Further, subjective and objective reactivity were greater to the TSST compared to the CO_2_ challenge. Additionally, significant cubic trends were observed in the interactions of stress framing and stress-induction methodologies on heart rate and blood pressure. Balanced framing conditions in the TSST group had a significantly larger decrease in heart rate and diastolic blood pressure following stress compared to the positive and negative framing conditions.

**Conclusion:**

Findings confirmed a deficit-orientation of stress within the sampled population. In addition, results highlighted the relative efficacy of the TSST compared to CO_2_ as a method of stress provocation. Finally, individuals in framing conditions that posited stress outcomes in unbalanced manners responded to stressors less efficiently. This suggests that unbalanced framing of stress may have set forth unrealistic expectations regarding stress that later hindered individuals from adaptive responses to stress. Potential benefits of alternative conceptualizations of stress on stress reactivity are discussed, and suggestions for future research are made.

## Introduction

The experience of stress has been known to diminish health, adjustment and well-being. Within the Transactional model of stress [1], stress is recognized as an interactive process between cognition and physiology and is defined in the current study as a stimulus, event, or situation that requires adaptation, and may challenge the existing equilibrium, whether psychologically or physically. As such, a stressful event can provoke a psychological and/or physiological response in various systems within an individual. Prolonged exposures to stress result in exhaustion of bodily resources, leaving one vulnerable to a myriad of mental and physical problems [2]. Thus, improving how individuals respond to stress has been the emphasis of much research.

The detrimental effects of stress on health outcomes are well recognized in studies and in literature reviews [2, 3]. It is also widely assumed that stress is negative, unpredictable, and threatening [4, 5], although this assumption has not yet been tested empirically. However, researchers are increasingly noting that the experience of stress can engender both positive and negative outcomes over time [6]. For example, successful coping with everyday stress could serve as a mechanism to motivate achievement, personal growth, self-confidence and coping self-efficacy. Indeed, even unsuccessful attempts at coping could facilitate motivation and an opportunity to challenge one’s skills.

Given that the consequences of stress can involve both positive and negative outcomes, actively (re)-framing what one perceives to be the outcomes of stressors may be key in facilitating adaptive responses to stress. Indeed, past studies have found that perceptions of the outcomes of stress are an important mediator in the relationship between stress and negative health outcomes [7]. Recent studies in stress [8–10] have successfully reduced stress responses by reframing the effects of anticipated stressors as more positive. However, the consequences of stress are hardly always positive. Consequently, a conceptualization of stress that is overly positive may not be beneficial in some contexts, such as when one’s views on stress are incongruent with reality [11]. For example, an overly positive message about stress might set forth expectations that one does not have to prepare for an upcoming event. This, coupled with the reality of experiencing an event that exerts demands on one’s resources, may then overwhelm the individual, hindering adaptive responses. Thus, it is important to examine how different expectations for the outcomes of stress may shape or affect individual reactivity to stress by first assessing a sampled population’s perceptions of stress, then manipulating these perceptions through different ways in which consequences of stress are framed.

A considerable literature has been dedicated to the validation and comparison of varied methods of stress-induction. Recent advances in the comparative effects of multiple methods of stress-induction have highlighted the Trier Social Stress Test (TSST) as a robust and well-validated method of provoking psychosocial stress [12, 13]. Other methods of engendering stress have received less empirical attention in comparison. To date, only a handful of studies have used the carbon dioxide test (a single vital capacity inhalation of air enriched with 35% carbon dioxide) as a stress provocation [14]. These preliminary findings suggest that the carbon dioxide (CO_2_) test may be a “very useful tool” ([15], p. 745) to provoke stress in the lab. Yet, no studies, to our knowledge, have compared the ability of the CO_2_ test, which can be thought of primarily as a physiological stressor, to engender the human stress response to that of a well-validated method of stress induction. Therefore, in the current research, we utilized both the CO_2_ test and the TSST, a reliable and robust method of stress-provocation.

Our research aims were as follows: to examine individuals’ beliefs about stress; to study whether these beliefs interact with how individuals respond to stress; and to determine whether the stress response can be influenced by different ways in which stress outcomes are framed. As noted above, we also sought to compare stress reactivity to the CO_2_ test and the TSST.

We hypothesized that the majority of the sampled population would conform to a negative perception of stress, and would believe that the consequences of stress are entirely negative. We also predicted that more positive beliefs about stress would facilitate reduced stressor reactivity via lower heart rate (HR), blood pressure (BP), and self-reported stress levels. We further hypothesized that by framing the effects of stress in a balanced manner to include both positive and negative information about stress outcomes, individuals would experience a faster recovery to baseline following stress, relative to framing conditions that described only the positive or the negative consequences of stress. Finally, in an exploratory fashion, we compared effects of the CO_2_ test on stress reactivity to that of the well-validated TSST psychosocial stressor.

## Methods

### Participants

A total of 97 participants successfully completed the study. Participants were enlisted through an Introductory Psychology Student Pool, which consists of a large cross-section of students from various disciplines, ethnicities, and years of study. Exclusion criteria were previous medical conditions such as asthma (that are contradicted with use of the CO_2_ test) and current levels of stress falling outside of two standard deviations of the population norm on the Perceived Stress Scale (PSS) [16]. Seven participants were removed following data cleaning; four participants were outliers, one participant’s physiological data was not available due to equipment malfunction, while two had missing data exceeding 20%.

The final sample size for all subsequent analysis thus consisted of 90 participants, with a mean age of 21 (SD = 4.53). Out of the 90 participants, 27 (30%) were males and 63 (70%) were females. Thirty-three percent self-identified as Caucasian, 23% identified as East Asian, 30% identified as South Asian, while others identified as mixed or other race/ethnicity.

### Materials and apparatus

#### 1. Framing of stress

Participants received one of three conceptualizations of stress via a 5-minute framing video: balanced, unbalanced negative, or unbalanced positive. To maximize experimental control and to ensure that only the content and wording was manipulated across videos, all three videos were shot in the same setting, featured the same actor, and were of the same length. The actor was a Caucasian female graduate student dressed in professional, business-casual attire, and maintained the same neutral but professional attitude throughout all three videos.

In the unbalanced-negative condition, the actor discussed the effects of stress by focusing on the negative effects of chronic stress, and advised viewers to engage in stress management to avoid these negative effects. The unbalanced-positive framing stress video focused selectively on the potential positive and adaptive effects of acute stress. Specifically, positive outcomes, such as increased motivation, vigilance, and efficient stress response times were highlighted. Finally, in the balanced framing stress video, both the negative and positive consequences of acute stress were discussed. In addition, the video emphasized an individual’s choices in determining one’s response to stress, and posited stress management as a muscle that needs constant exercising in order to foster resiliency.

#### 2. Methods of stress induction

Participants were randomized to undergo one of two stress-induction methods: the CO_2_ test [14] or the TSST [17]. The CO_2_ test has been extensively used in past research to as a potent panicogen in those with panic disorder. In healthy participants, the CO_2_ test has been advocated as a stress task [14] due to its induction of physiological changes along with mild subjective anxiety [14, 18]. The CO_2_ test involves a single vital-capacity breath of 35% CO_2_ and 65% oxygen that is held for four seconds prior to exhalation.

The TSST is a widely used laboratory test to induce stress in both clinical and healthy participants [17]. The TSST consists of an anticipation/speech-preparation period, a speech delivery period in which participants speak on a topic, and a task period involving a backwards subtraction task, each task lasting ten minutes. To match the duration of the CO_2_ challenge, the TSST protocol was shortened to five minutes per task in the current study, similar to the modifications made in Skoluda et al.’s study [13].

### Measures

#### 1. Demographics information

Basic demographic information, such as gender, age, and ethnicity was collected at the beginning of the study.

#### 2. Perception of stress

To gauge whether the sample indeed had a deficit-orientation of stress, a free association task was used in which participants were asked to name up to four words associated with ‘stress’. This task is a reliable and commonly used tool to index existing knowledge, norms, and perceptions on a given topic [19].

#### 3. Physiological stress response

Objective indications of stress responsivity consisted of heart rate (HR), systolic, and diastolic blood pressure (SBP; DBP) from the Biopac CNAP^®^ blood pressure system, a continuous, non-invasive measure of heart rate and blood pressure. Data were time-period marked for five time-points, including baseline one (t1; questionnaire period, approximately five to ten minutes in length), framing (t2; video period, five minutes in length), baseline two (t3; post-video questionnaire period, three minutes), stressor (t4; during either TSST or CO_2_ task period, both 15 minutes), and post-stressor (t5; recovery and questionnaire period, approximately five to ten minutes).

#### 4. Subjective stress response

A Visual Analogue Scale (VAS) assessing for levels of stress was given to participants to rate their current levels of stress immediately following the stress-induction task. The scale asks participants to rank their current levels of stress, such that “0 means no stress or discomfort, where you do not feel disturbed at all, and 100 means extreme stress, or the worst kind of distress experienced imaginable”. The scale is a valid and reliable instrument for measuring characteristics and values along a continuum, and ranged from 0 (not at all stressed) to 100 (extremely stressed) [20].

### Procedure

Participants were invited for a 60-minute session at Ryerson University. They were randomly assigned to one of three framing conditions: (1) balanced framing condition (*n* = 30), (2) unbalanced-negative framing condition (*n* = 30), and (3) unbalanced-positive framing condition (*n* = 30). In addition, participants were also randomly assigned to one of two stress-induction conditions: (a) the Trier Social Stress Test (*n* = 45), and (b) the CO_2_ challenge (*n* = 45). Randomization resulted in fifteen participants assigned per group, for a total of six mutually-exclusive experimental groups. Testing for participants occurred between 9am to 5pm on weekdays. Written informed consent was obtained from participants prior to the beginning of the study. This study was approved by Ryerson University's Research Ethics Board (REB) in Toronto, Canada (REB 2014–188).

Upon arrival, each participant was connected to the Biopac Continuous Non-invasive Arterial Pressure (CNAP)^®^ NIBP100D blood pressure system. His or her non-dominant arm was used for measures of blood pressure and heart rate via a finger clip transducer for pulse. Participants then answered a series of questions, and watched a 5-minute framing video on stress. Next, participants underwent one of two forms of stress-induction. Lastly, participants completed additional questions, including the VAS measure of subjective stress reactivity, and were disconnected from the CNAP machine, debriefed and compensated via course credit for their participation.

### Statistical analysis

All quantitative analyses were conducted using IBM’s Statistical Package for the Social Sciences (SPSS) Software^®^, version 21.0. The interactive effects of both experimental manipulations, including stress framing and stress-induction, were assessed via mixed-ANOVA, with specific emphasis on trend analysis. A post hoc power analysis using G*Power for a mixed ANOVA (five by three by two interaction) design with an alpha of .05 was found to have a statistical power of 0.72 to detect an effect sizes of 0.25, considered a small to medium effect under Cohen’s guidelines [21].

## Results

### Participant stress orientation and perception

To gauge whether the sample indeed had a deficit-oriented approach to understanding the consequences of stress [5], two blind raters were asked to categorize each response from the free association task as positive (1), negative (-1), or neutral (0). The inter-rater reliability was 84.26% overall, and conflicts were resolved through discussion. Chi-square goodness-of-fit test determined whether negative, positive, and neutral orientations were equally rated amongst participants. Results revealed that stress orientation among participants were not equally distributed in the sampled population, *X*^*2*^ (2, *N* = 90) = 53.07, *p* < .001. The majority (66.67%) of participants rated the first word associated with stress as negative (see Table 1). Further, the overall ratings were also predominantly negative, with 76.47% of participants associating stress with more negative than positive words, while only 16.47% of participants considered stress to be more positive than negative.

**Table 1:** Ratings of stress via word association task.

	Frequency	Percent	Cumulative Percent
**Negative**	60	66.7	66.7
**Neutral**	26	28.9	95.6
**Positive**	4	4.4	100.0

Table represents the proportions of the sampled population whose first word to describe stress was negative, neutral, or positive.

### Prior beliefs of stress on subjective reactivity to stress

In order to determine if prior beliefs on stress may serve to confound later subjective responses to stressors, a one-way analysis of variance examined whether participants’ stress orientation affected outcomes on the VAS. Due to the large differences in group sizes between negative (*n* = 60), neutral (*n* = 26), and positive (*n* = 4), we pooled the neutral and positive into a single group. Tests of between-subjects effects did not reveal significant differences in subjective stress responses based on initial perceptions of stress: *F*(1, 88) = .311, *p* = .579, *η*^2^ = .004. Participants’ subjective responses to stress were similar despite initial perceptions of stress as negative (subjective stress response *M* = 41.02, *SD* = 27.88), or neutral/positive (*M* = 37.63, *SD* = 25.57).

### Experimental manipulations on subjective stress reactivity

A three-by-two analysis of variance with framing condition (Negative, *n* = 30, Balanced, *n* = 30; Positive, *n* = 30) and stress induction (TSST, *n* = 45; CO_2_, *n* = 45) as between-subject factors examined whether these experimental manipulations contributed to differences in subjective stress reactivity.

**Fig 1:**
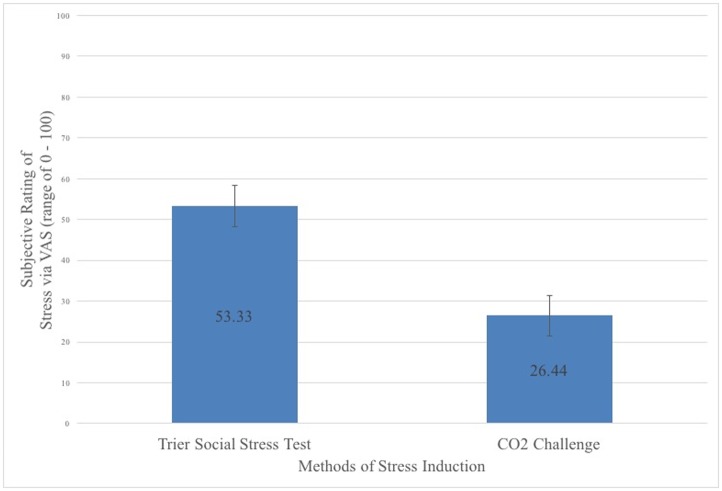
Subjective stress responses across methods of stress induction. Figure illustrates the comparative efficacy of a well-validated method of stress induction and a novel method of physiological stress induction on self-reported ratings of stress. Error bars represent the standard error for each method of induction.

Findings did not reveal a significant effect of framing condition on participants’ subjective response to stress: *F*(2, 84) = 1.287, *p* = .282, *η*^2^ = .03. Participants’ subjective responses to stress were similar across the negative framing (*M* = 40.83, *SD* = 29.71), balanced framing (*M* = 34.39, *SD* = 23.10), and positive framing (*M* = 44.79, *SD* = 27.89) conditions. However, results revealed a significant effect of stress condition on participants’ subjective response to stress: *F*(1, 84) = 29.29, *p*< .001, *η*^*2*^ = .26. Compared to those that underwent the CO_2_ stress induction (*M* = 26.44, *SD* = 22.87), those that underwent the TSST reported a significantly higher subjective stress response (*M* = 53.33, *SD* = 24.21) (see Fig 1). The interactive effects of stress framing and stress induction did not significantly affect subjective stress reactivity: *F*(2, 84) = .908, *p* = .407, *η*^*2*^ = .02.

### Experimental manipulations on objective measures of stress reactivity

To examine differences in physiological stress parameters across experimental groupings, a five-by-three-by-two (5x3x2) mixed-measures ANOVA was used to examine the effects of time (from baseline to recovery) on different objective measures of stress (HR, SBP, DBP). The two between-subject factors for this ANOVA were framing condition (Negative, Balanced, Positive) and stress-induction condition (TSST, CO_2_). All physiological data were Log transformed (Log^10^) prior to analyses to normalize the data.

Mauchly’s test indicated that the assumption of sphericity was violated for all three physiological stress parameters: heart rate (*χ*^2^(9) = 133.114, *p* <.001), systolic blood pressure (χ^2^(9) = 2066.06, *p* <.001), and diastolic blood pressure (*χ*^*2*^(9) = 146.975, *p* <.001); therefore, degrees of freedom were corrected using Greenhouse-Geisser estimates of sphericity (ε = 0.551 for heart rate; ε = 0.250 for systolic blood pressure; ε = 0.530 for diastolic blood pressure).

**Fig 2:**
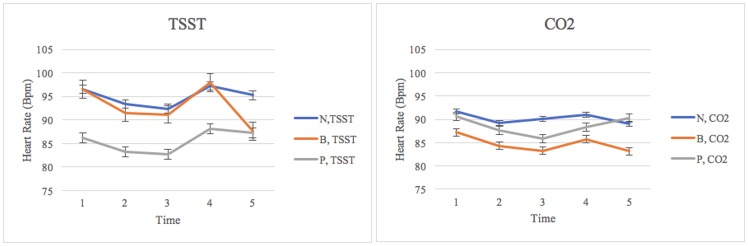
Group differences in heart rate over time. *N = Negative Framing; B = Balanced Framing; P = Positive Framing; Time 1 = baseline; Time 2 = framing video; Time 3 = baseline 2/rest; Time 4 = stressor; Time 5 = stress response/recovery; TSST = Trier Social Stress Test; CO2 = carbon dioxide challenge. Figure illustrates the heart rate trajectories of experimental groups across the entirety of the experiment.

Thus, after adjusting for degrees of freedom, results from the mixed-ANOVA indicated a significant interaction between cubic time and framing on heart rate: *F*(4.41, 185.23) = 3.09, *p* = .01, *η*^*2*^ = .07. Within-subject contrasts revealed a significant cubic interaction between time, framing condition, and stressor on measures of heart rate: *F*(2, 84) = 27.53, *p* = .048, *η*^2^ = .07. Figure two illustrates group differences in heart rate over time using untransformed data (Fig 2).

Post hoc analyses using a five-by-three (5x3) mixed ANOVA independently examined the effects of stressor and framing on HR across different time points. Results revealed that group differences across two stressors were approaching significance during t4 (stressor): *F*(1, 88) = 3.81, *p* = .054. During the stressor, the combined heart rate in the TSST group (*M* = 94.44, *SD* = 17.49) was higher than that of the CO_2_ groups (*M* = 88.34, *SD* = 11.54). However, framing conditions did not result in significant group differences in HR across time points (see Table 2).

**Table 2:** Post-hoc analyses of heart rate via one-way ANOVA.

	Time	*F*	*df*	Sig.
**Stressor**				
	Baseline	1.22	1, 88	.27
	Video	0.65	1, 88	.42
	Baseline 2	0.58	1, 88	.45
	Stressor	3.81	1, 88	.05
	Recovery	0.57	1, 88	.45
**Framing**				
	Baseline	0.12	2, 87	.90
	Video	0.17	2, 87	.68
	Baseline 2	0.21	2, 87	.65
	Stressor	0.02	2, 87	.88
	Recovery	2.67	2, 87	.11

*F* represents the F-ratio statistics; *df* represents degrees of freedom; *Sig*. represents the significance level (p <.05). Table represents the results from a one-way analysis of variance examining the effects of experimental manipulations on diastolic blood pressure at each time point between groups. Table represents the results from a one-way analysis of variance examining the effects of experimental manipulations on heart rate at each time point between groups.

To ensure that the effects of framing were not masked by the larger effects of the stressor types, we conducted separate post hoc analyses using a repeated one-way ANOVA for framing condition across different time points for both the TSST and the CO_2_ groups. While results indicate that there was not a significant time and framing interaction for the CO_2_ group, *F*(8, 80) = .46, *p* = .46, *η*^*2*^ = .09, there was a significant time and framing condition interaction for the TSST group, *F*(8, 80) = 2.42, *p* = .02, *η*^*2*^ = .19. To further investigate this interaction within the TSST group, change scores were computed for changes in heart rate from stressor to recovery, and a one-way ANOVA comparing different framing conditions indicated a significant effect of framing *F*(2, 42) = 8.78, *p* = .001. Post hoc comparisons indicate that the balanced framing condition experienced a greater decline in heart rate from stressor to recovery (*M* = -10.31, *SD* = 3.61) compared to both the positive framing condition (*M* = -.95, *SD* = 9.89) and the negative framing condition (*M* = -2.04, *SD* = 4.87).

**Fig 3:**
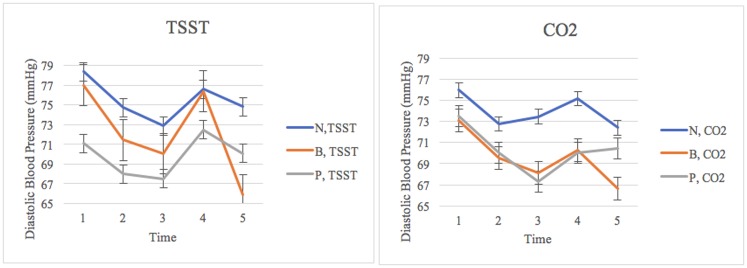
Group differences in diastolic blood pressure over time. *N = Negative Framing; B = Balanced Framing; P = Positive Framing; Time 1 = baseline; Time 2 = framing video; Time 3 = baseline 2/rest; Time 4 = stressor; Time 5 = stress response/recovery; TSST = Trier Social Stress Test; CO2 = carbon dioxide challenge. Figure illustrates the diastolic blood pressure trajectories of experimental groups across the entirety of the experiment.

Results from the repeated measures ANOVA also indicated a significant interaction between cubic time and framing on diastolic blood pressure: *F*(4.24, 178.12) = 3.33, *p* = .01, *η*^*2*^ = .07. Within-subject contrasts also revealed significant trends. As determined by the largest effect size, a significant cubic interaction trend between time, framing condition, and stress induction was observed for diastolic blood pressure, *F*(2, 84) = 4.76, *p* = .01, *η*^2^ = .102. Figure three illustrates group differences in diastolic blood pressure over time using untransformed data (Fig 3).

Post hoc analyses using a one-way ANOVA independently examined the effects of stressor and framing on DBP across different time points. While results did not reveal the existence of group differences across two stressors, the analysis of variance revealed that framing conditions resulted in group differences in DBP during t5 (recovery) approaching statistical significance: *F*(1, 87) = 3.33, *p* = .07. During recovery, the combined group mean for the balanced framing were observed to be the lowest (*M* = 66.29, *SD* = 13.75), compared to the group receiving the positive framing (*M* = 70.25, *SD* = 12.87), and negative framing (*M* = 73.61, *SD* = 15.07). However, post-hoc comparisons did not detect any significant group differences (see Table 3).

**Table 3:** Post-hoc analyses of diastolic blood pressure via one-way ANOVA.

	Time	*F*	*df*	Sig.
**Stressor**				
	Baseline	0.26	1, 88	.61
	Video	0.06	1, 88	.81
	Baseline 2	0.04	1, 88	.85
	Stressor	1.56	1, 88	.21
	Recovery	0.03	1, 88	.86
**Framing**				
	Baseline	.01	2, 87	.93
	Video	.12	2, 87	.73
	Baseline 2	.21	2, 87	.65
	Stressor	.02	2, 87	.90
	Recovery	3.33	2, 87	.07

*F* represents the F-ratio statistics; *df* represents degrees of freedom; *Sig*. represents the significance level (p <.05). Table represents the results from a one-way analysis of variance examining the effects of experimental manipulations on diastolic blood pressure at each time point between groups. Table represents the results from a one-way analysis of variance examining the effects of experimental manipulations on heart rate at each time point between groups.

Additional post hoc analyses using a five-by-three (5x3) mixed ANOVA unmasked the interactions of framing on diastolic blood pressure across different time points for both the TSST and the CO_2_ groups. While results indicate that there was not a significant time and framing interaction for the CO_2_ group, *F*(8, 80) = 1.47, *p* = .18, *η*^*2*^ = .13, there was a significant time and framing condition interaction for the TSST group, *F*(8, 80) = 2.99, *p* = .005, *η*^*2*^ = .23. To further investigate this interaction within the TSST group, change scores were computed for changes in diastolic blood pressure from stressor to recovery, and a one-way ANOVA comparing different framing groups indicated a significant effect of framing *F*(2, 42) = 9.93, *p* = <.001. Post hoc comparisons indicate that the balanced framing condition experienced a greater decline in diastolic blood pressure from stressor to recovery (*M* = -10.50, *SD* = 2.70) compared to both the positive framing condition (*M* = -2.39, *SD* = 7.82) and the negative framing condition (*M* = -1.76, *SD* = 6.27).

Findings from the within-subject contrasts discussed detected no significant effects on participants’ systolic blood pressure: *F*(1,84.03) = 1.00, *p* = .32, *η*^*2*^ = .012. Likewise, within-subject contrasts also did not detect any significant trends *F*(2, 84) = 1.01, *p* = .37, *η*^2^ = .02.

## Discussion

The present study examined the interaction(s) between perceptions of stress, framing of stress outcomes, and methods of stress induction on individual stress responsivity. Based on reviewed evidence suggesting a deficit-orientation of stress in both the research literature and public perception [5], we postulated that a deficit-orientation of stress would be normative in the sample. Indeed, the findings were in line with our hypothesis. Results from the qualitative content analysis confirmed a deficit-orientation of stress in the sample, with a large majority associating stress with negative words. Interestingly, there was no effect of individual associations of stress on later subjective reactivity. Initial beliefs of stress as negative, positive, or neutral did not affect how participants later responded to methods of stress induction. However, this may be attributed to the large majority of the sampled population already subscribing to a deficit-orientation of stress, thereby resulting in a homogenous sample.

Results revealed a relatively low potency of CO_2_ as a method of stress-induction compared to the TSST. Subjective and objective indicators of stress reactivity emphasized the differential effects of the methods of stress-induction. Though exploratory, these finding are in line with existing literature suggesting that a psychosocial stressor is a more robust method of stress induction as compared to a physiological stressor [13]. The differences in physiological and psychological responses to the CO_2_ in comparison to the TSST could be attributed to some individuals failing to respond to the CO_2_ challenge as a stressor altogether. Indeed, although some previous research has established that the CO_2_ challenge provokes some degree of physiological stress responses in healthy individuals [22], studies have also documented a lack of reactivity in some healthy individuals, subsequently categorized as ‘non-responders’ to the CO_2_ challenge [15].

The CO_2_ challenge has traditionally been studied in clinical populations, as characterized by the provocation of panic-like symptoms in individuals with a history of panic or anxiety disorders [14, 18] or other psychiatric conditions [23]. The variable responses of individuals to the CO_2_ test may be attributed to differences in cognitive appraisal and tolerance of physiological symptoms between healthy populations and clinical populations with a history of anxiety or panic disorders. Future studies could compare the relative potency and efficacy of the CO_2_ stress-induction and strength of physiological and psychological responses across both healthy and clinical populations.

### The importance of expectations

Much of the mental health movement has been pre-occupied with negative outcomes of stress, and thus has emphasized deficit-orientation in the approach to the consequences of stress [24]. Similarly, the current findings suggest that the view of stress in the layperson also has a deficit orientation. Although anticipating and preparing for stress may be helpful in alerting individuals to threats, preoccupation with stress may also result in fear, catastrophizing, and avoidance altogether.

We hypothesized that balanced framing of stress outcomes would be conducive to healthy, adaptive responses to stressors. Post hoc analyses following the significant cubic interaction between time, stressor, and framing condition revealed that participants in the balanced framing conditions that underwent the TSST experienced the most efficient declines in physiological arousal following the stressor, which is a demonstrated sign of cardiac efficiency and resilience [10]. This is especially compelling given the lack of baseline differences across various measures at baseline. Compared to other framing conditions, providing an individual with both positive and negative information about stress outcomes may help prevent unrealistic or biased expectations regarding stressors.

The larger declines in heart rate and diastolic blood pressure during recovery experienced by the balanced framing group undergoing the TSST, in comparison to other groups, support the importance of the expectations regarding stress and recovery. By presenting realistic information on stress and the effects of stress, individuals may have felt more informed and autonomous in their own psychological and physiological responses to stress. These expectations set forth by a video prior to the experience of stress may have changed the physiological response trajectory of the individual stress response, facilitating a quicker, more efficient recovery. The balanced framing conditions allowed the individual to process and cope with the stressor in an efficient and adaptive manner. As stress is unavoidable in daily life, the emphasis should not be to change the stressor, but rather to promote more adaptive coping through education and management of individual expectations in responses to stress.

On the other hand, if the information given in a framing condition were to set forth specific expectations regarding stress, and those expectations are violated or became incongruent upon facing a stressor, then the violation of expectations could potentially augment distress for the participant. Perhaps the positive framing set forth expectations that stress is easy to cope with. This, coupled with the demands of the TSST, could overwhelm the participants as reality violated the expectations set by the framing video.

Telch et al experimentally induced unexpected arousal versus expected arousal, and found the violation of expectations contributed to increased vulnerability and reactivity to the CO_2_ challenge [11]. Gruber also discussed the downside of positive emotions in his research. His findings imply that positivity experienced in the wrong context could potentially negate any benefits from feeling positive [25]. The analogy of a mismatch between expectations and reality could explain the interactive effects of experimental manipulations on stress physiology. Positive framing and expectations, coupled with an intense stressor, could violate expectations and contribute to further exacerbate the stress experienced. On the other hand, overly negative framing and expectations of stress coupled with a less intense stressor could result in decreased stress reactivity.

### Implications and limitations

Findings from the current study contribute to a growing body of literature that examines the efficacy of re-appraisal-based interventions that are targeted to improve stress reactivity [8–10, 26–28]. The current study was novel in that it targeted the deficit-orientation in conceptualizations of stress, and introduced alternative conceptualizations that challenged this deficit-orientation. Further, our study findings also lend support to the importance of managing one’s perceptions and expectations in stress. Information presented regarding stress, coupled with the type of stressor subsequently encountered, both play key roles in the response to stress. If the information given in a framing condition sets forth specific expectations regarding stress, and those expectations are violated or become incongruent upon facing a particular stressor, then the violation of expectations could potentially augment distress for the participant.

The individualistic nature of stress is a challenge and potential limitation within the current study. A shortcoming of our design is the lack of a control group, which limits the interpretation of our results. The relatively low responses to CO_2_ across experimental groups may indicate that CO_2_ may not be a reliable and robust method to provoke psychological stress amongst healthy populations. However, physiological data suggest that despite lowered reactivity in comparison, participants still responded, to some degree, to the CO_2_ as a method of stress induction. Further, we did not pilot test the framing videos and their contents. Despite taking two baseline measures to ensure that the video did not confound with arousal from stressors, we did not measure what viewers thought of each video and its contents. As such, the interpretation of the interactive effects of the framing manipulation need to be conservatively made.

In addition, participants in this study may be homogeneous in their relative exposures to stress, as well as perceptions of stress. Past exposure to stress is an important factor in shaping future responses and coping with stress [29]. Although results did not find that stress orientation affected individual responses to stress, the group sizes for this analysis were different, and therefore conclusions drawn from this finding should be interpreted cautiously. Thus, future research should account for these differences to retain better control of the experimental manipulations.

Finally, based on the disproportionate sex distribution in the sampled population, we did not conduct analyses to compare sex differences. Past research has highlighted the existence of sex differences in stress reactivity [30, 31]. However, uneven sampling and overrepresentation of females in the current sample prevented us from examining whether sex differences interacted with our experimental manipulations. Further, we did not control for individual characteristics, such as contraceptive use or menstrual cycle in our sampled population. Thus, findings of the current study should be taken with consideration that sex differences may not be fully represented.

The current study also has unique strengths. Although previous research has highlighted the deficit-orientation in stress conceptualization, this is the first study to directly challenge the different conceptualizations of stress using a framing video manipulation. Going one step beyond past research that has utilized reappraisal to improve stress and coping, the current study introduced different conceptualizations of stress in order to gain a better understanding of how our thoughts regarding stress may influence or restructure future coping with different stressors.

Although attempts to reduce stress should never be overlooked by an individual or the healthcare system, when stress inevitably occurs, a more balanced understanding, a strengthened coping efficacy, diverse stress management skills and enhanced resilience may be useful for individuals in approaching non-traumatic stress as a challenge, and in potentially experiencing growth and resiliency from these beliefs.

## Supporting information

S1 Videos Balanced (B), Unbalanced-Positive (UBP), and Unbalanced-Negative (UBN) framing videos. This compressed file includes the three framing videos included in our experimental manipulations.
(ZIP)

## References

[1] Lazarus RS, Folkman S. Stress, appraisal, and coping. New York, NY: Springer; 1984.

[2] Stratakis CA, Chrousos GP. Neuroendocrinology and pathophysiology of the stress system. Annals of the New York Academic of Sciences. 1995; 771: 1–18. 10.1111/j.1749-6632.1995.tb44666.x 8597390

[3] Wolkowitz OM, Rothschild AJ. Psychoneuroendocrinology. The scientific basis of clinical practice. Washington, DC: American Psychiatric Publishing 2003.

[4] Lupien S. Well stressed: Manage stress before it turns toxic. Mississauga, Ontario: John Wiley & Sons Canada 2012.

[5] Liu JJW, Vickers K. New developments in stress research–Is stress all that bad? New evidence for mind over matter In: Columbus AM, editor. Advances in psychology research, 106th ed Hauppauge, NY: Nova Publishers; 2015 pp. 125–136.

[6] Harrington R. (2013). Stress, health & well-being: Thriving in the 21st century. Belmont, CA: Wadsworth, CENGAGE Learning.

[7] Keller A, Litzelman K, Eisk LE, Maddox T, Cheung ER, Creswell PD, et al Does the perception that stress affects health matter? The association with health and mortality. Health Psychology. 2012; 31: 677–681. 10.1037/a0026743 22201278 PMC3374921

[8] Jamieson JP, Mendes WB, Blackstock E, Schmader T. Turning the knots in Your stomach into bows: Reappraisal arousal improves performance on the GRE. Journal of Experimental Social Psychology. 2010; 46: 208–212. 10.1016/j.jesp.2009.08.015 20161454 PMC2790291

[9] Jamieson JP, Nock MK, & Mendes WB. Changing the conceptualization of stress in social anxiety disorder: Affective and physiological consequences. Clinical Psychological Science. 2013; 1: 363–374.

[10] Jamieson JP, Nock MK, Mendes WB. Mind over matter: Reappraising arousal improves cardiovascular and cognitive responses to stress. Journal of Experimental Psychology: General. 2012; 141: 417–422. 21942377 10.1037/a0025719 PMC3410434

[11] Telch MJ, Harrington PJ, Smits JAJ, Powers MB. Unexpected arousal, anxiety sensitivity, and their interaction on the CO_2_-induced panic: Further evidence for the context-sensitivity vulnerability model. Journal of Anxiety Disorder. 2011; 25: 645–653. 10.1016/j.janxdis.2011.02.005 21474277

[12] Dickerson SS, Kemeny ME. Acute stressors and cortisol responses: A theoretical integration and synthesis of laboratory research. Psychological Bulletin. 2004; 130: 355–391. 10.1037/0033-2909.130.3.355 15122924

[13] Skoluda N, Strahler J, Schlotz W, Niederberger L, Marques S, Fischer S, et al Intra-individual psychological and physiological responses to acute laboratory stressors of different intensity. Psychoneuroendocrinology. 2015; 51: 227–236. 10.1016/j.psyneuen.2014.10.002 25462896

[14] Kaye J, Buchanan F, Kendrick A, Johnson P, Lowry C, Bailey J, et al Acute carbon dioxide exposure in healthy adults: Evaluation of a novel means of investigating the stress response. Journal of Neuroendocrinology. 2004; 16: 256–264. 10.1111/j.0953-8194.2004.01158.x 15049856

[15] Wetherell MA, Crown AL, Lightman SL, Miles JNV, Kaye J, Vedhara K. The four-dimensional stress test: Psychological, sympathetic-adrenal-medullary, parasympathetic and hypothalamic-pituitary-adrenal responses following inhalation of 35% CO_2_. Psychoneuroendocrinology. 2006; 31: 736–747. 10.1016/j.psyneuen.2006.02.005 16621326

[16] Cohen S, Kamarack T, Mermelstein R. A global measure of perceived stress. Journal of Health and Social Behavior. 1983; 24: 386–396. Retrieved from http://www.ncbi.nlm.nih.gov/pubmed/6668417 6668417

[17] Kirschbaum C, Pirke K, Hellhammer DH. The ‘Triers Social Stress Test’–a tool for investigating psychobiological stress responses in a laboratory setting. Neuropsychobiology. 1993; 28: 76–81. Retrieved from http://www.ncbi.nlm.nih.gov/pubmed/8255414 8255414 10.1159/000119004

[18] Vickers K, Jafarpour S, Mofidi A, Rafat B, Woznica A. The 35% carbon dioxide test in stress and panic research: Overview of effects and integration of findings. Clinical Psychology Review. 2012; 32: 153–164. 10.1016/j.cpr.2011.12.004 22322014

[19] Nelson DL, McEvoy CL, Schreiber TA. The University of South Florida free association, rhyme, and word fragment norms. Behavioral Research Methods, Instruments, & Computers. 2004; 36: 402–407. 10.3758/bf03195588 15641430

[20] Wewers ME, Lowe NK. A critical review of visual analogue scales in the measurement of clinical phenomena. Research in Nursing and Health. 1990; 13: 227–236. 2197679 10.1002/nur.4770130405

[21] Cohen J. Statistical power analysis for the behavioral sciences. New York, NY: Routledge; 1988.

[22] van Duinen MA, Schruers KRJ, Maes M, Griez EJ. CO_2_ challenge results in hypothalamic-pituitary-adrenal activation in healthy volunteers. Journal of Psychopharmacology. 2005; 19: 243–247. 10.1177/0269881105051527 15888509

[23] Woznica A, Vickers K, Koerner N, Fracalanza K. Reactivity to 35% carbon dioxide in bulimia nervosa and panic disorder. Psychiatry Research. 2015; 228: 571–575. 10.1016/j.psychres.2015.05.050 26141602

[24] Seligman MEP, Csikszentmihalyi M. Positive Psychology: An introduction. American Psychologist. 2000; 55: 5–14. 11392865 10.1037//0003-066x.55.1.5

[25] Gruber J. Can feeling too good be bad? Positive Emotion Persistence (PEP) in Bipolar Disorder. Current Directions in Psychological Science. 2011; 20: 217–221.

[26] Beltzer ML, Nock MK, Peters BJ, Jamieson JP. (2014). Rethinking butterflies: The affective, physiological, and performance effects of reappraising arousal during social evaluation. Emotion. 2011; 14: 761–765. 24749642 10.1037/a0036326

[27] Cohen GL, Garcia J, Apfel N, Master A. Reducing the racial achievement gap: A social-psychological intervention. Science. 2006; 313: 1307–1312. Retrieved from www.sciencemag.org 10.1126/science.1128317 16946074

[28] Schmidt NB, Eggleston AM, Woolaway-Bickel K, Fitzpatrick KK, Vasley MW, Richey JA. Anxiety sensitivity amelioration training (ASAT): A longitudinal primary prevention program targeting cognitive vulnerability. Journal of Anxiety Disorders. 2007; 21: 302–319. 10.1016/j.janxdis.2006.06.002 16889931

[29] Michie S. Causes and management of stress at work. Occupational and Environmental Journal. 2002; 59: 67–72. 10.1136/oem.59.1.67 PMC1740194 11836475

[30] Kelly MM, Tyrka AR, Anderson GM, Price LH, Carpenter LL. Sex differences in emotional and physiological response to the Triers Social Stress Test. Journal of Behavioral Theory and Experimental Psychiatry. 2008; 39: 87–98. 10.1016/j.jbtep.2007.02.003 PMC4467692 17466262

[31] Taylor SE, Klein LC, Lewis BP, Gruenewald TL, Gurung RAR, Updegraff JA. Biobehavioral responses to stress in females: Tend-and-befriend, not fight-or- flight. Psychological Review. 2000; 107: 411–429. 10941275 10.1037/0033-295x.107.3.411

